# Novel MXene-Modified Polyphenyl Sulfone Membranes for Functional Nanofiltration of Heavy Metals-Containing Wastewater

**DOI:** 10.3390/membranes13030357

**Published:** 2023-03-20

**Authors:** Mohammed Azeez Naji, Hamed Salimi-Kenari, Qusay F. Alsalhy, Raed A. Al-Juboori, Ngoc Huynh, Khalid T. Rashid, Issam K. Salih

**Affiliations:** 1 Faculty of Engineering and Technology, University of Mazandaran, Babolsar 4741613534, Iran; 2 Membrane Technology Research Unit, Chemical Engineering Department, University of Technology-Iraq, Alsinaa Street 52, Baghdad 10066, Iraq; 3 NYUAD Water Research Centre, New York University Abu Dhabi Campus, Abu Dhabi P.O. Box 129188, United Arab Emirates; 4 Department of Bioproducts and Biosystems, School of Chemical Engineering, Aalto University, P.O. Box 11000, Aalto, FI-00076 Espoo, Finland; 5 Department of Chemical Engineering and Petroleum Industries, AlMustaqbal University College, Babylon 51001, Iraq

**Keywords:** nanofiltration, nanocomposite membrane, wastewater, heavy metals, MXene

## Abstract

In this work, MXene as a hydrophilic 2D nanosheet has been suggested to tailor the polyphenylsulfone (PPSU) flat sheet membrane characteristics via bulk modification. The amount of MXene varied in the PPSU casting solution from 0–1.5 wt.%, while a series of characterization tools have been employed to detect the surface characteristics changes. This included atomic force microscopy (AFM), scanning electron microscopy (SEM), contact angle, pore size and porosity, and Fourier-transform infrared spectroscopy (FTIR). Results disclosed that the MXene content could significantly influence some of the membranes’ surface characteristics while no effect was seen on others. The optimal MXene content was found to be 0.6 wt.%, as revealed by the experimental work. The roughness parameters of the 0.6 wt.% nanocomposite membrane were notably enhanced, while greater hydrophilicity has been imparted compared to the nascent PPSU membrane. This witnessed enhancement in the surface characteristics of the nanocomposite was indeed reflected in their performance. A triple enhancement in the pure water flux was witnessed without compromising the retention of the membranes against the Cu^2+^, Cd^2+^ and Pd^2+^ feed. In parallel, high, and comparable separation rates (>92%) were achieved by all membranes regardless of the MXene content. In addition, promising antifouling features were observed with the nanocomposite membranes, disclosing that these nanocomposite membranes could offer a promising potential to treat heavy metals-containing wastewater for various applications.

## 1. Introduction

The massive wastewater amounts discharged into the aquatic system are becoming an increasingly significant concern threatening the ecosystem. The toxic nature of some contaminants in the wastewater, such as heavy metal ions, produced by some industrial activities can adversely affect marine life, plants and human health [1,2]. Therefore, there is a necessity to eliminate/minimize these contaminants to an allowable limit before discharge. So far, unlimited efforts have been devoted to conducting an efficient, simple and economic removal of heavy metals. Adopted techniques manifested a disparate degree of success depending on complex circumstances (e.g., the wastewater composition, required water quality, heavy metal level and stream size) [3,4,5,6,7,8,9,10,11,12].

Among the realistic treatment methods, membrane separation endows limitless opportunities to separate these hazardous heavy metal ions at minimal economic and environmental costs [13,14,15]. In this context, a tremendous amount of systematic scientific research has been devoted to getting deep insights into enhancing membrane performance. This was carried out through playing with fabrication parameters aiming to obtain a membrane with desired selectivity and permeation characteristics. These parameters are directly interlinked with membrane characteristics such as hydrophilicity, pore size and distribution, roughness and surface charges. However, the intrinsic hydrophobic nature of polymeric membranes makes them prone to rapid fouling during industrial operations [16]. Revising the surface characteristics of these membranes through tailoring their hydrophilicity has been believed as the bottleneck to overcoming membrane fouling. This indeed necessitates a functional modification to impart better hydrophilicity characteristics at the surface of the membrane [17,18,19,20,21,22,23,24,25,26,27]. In this context, limitless modification techniques employing versatile techniques and additives have been suggested.

With the rapid growth in nanotechnology and its outstanding applications, various novel nanoscale materials (0D, 1D and 2D) have simply found their way into water treatment applications. The nanoscale materials were extensively harnessed for versatile wastewater treatment applications as a standalone or nanocomposite adsorbents [28]. Their low cost, availability and promising potential to eliminate various contaminants have contributed to the circular economy [29]. In addition, materials at the nanoscale level have been utilized to impart specific desired features at membrane internal structures and surfaces, such as hydrophilicity, porosity, antimicrobial and self-cleaning. For the trace-heavy metals’ elimination from water, vacuum enhanced-membrane distillation via PVDF/TiO_2_ electrospun hybrid membranes was carried out by Moradi et al. (2016) [30]. Having a 2D structure composed of carbonitrides, nitrides and carbides, MXene (Ti_3_C_2_T_x_) nanosheets are promising materials with exceptional potential for the future of material science [31]. This structure has given rise to remarkable mechanical, chemical and physical properties. Parallel to that, with the versatile hydrophilic functional groups such as oxygen (O), hydroxyl (OH) and fluorine (F), MXene exhibits a highly hydrophilic nature and specific surface area [32,33]. Depending on the application, MXene could be synthesized with tailored characteristics [34]. MXene and its composites have been employed in a wide spectrum of applications including catalysis and photo electrocatalysis [35], medical and biomedical [36], lithium- and sodium-ion batteries [37], energy harvesting [38], antimicrobial [39], supercapacitors and hybrid capacitors [40]. Parallel to this steady growth in MXene applications, MXene has easily found a massive interest in water and wastewater treatment applications. Imparting MXenes’ hydrophilic functional groups onto the surface of the polymeric membrane could enhance the permeation and separation characteristics of these membranes. During the past decade, MXene has been widely employed in the separation field as a standalone membrane. However, the lack of MXene membrane stability is a big concern due to the oxidation and swelling of MXene in water [41]. Many attempts were conducted to overcome this issue. Liu et al. (2022) incorporated ascorbic acid into the body of the MXene structure and crosslinked the membrane layers with aluminum ions [42]. The membrane showed better anti-swelling behavior with 99.3% retention against dyes. Additionally, the transmembrane passage of salts (MgCl_2_ and NaCl) was minimized during the forward osmosis process. Wang et al. (2021) employed a laminar hydroxylated MXene membrane for separating heavy metal cations (Pb^2+^, Cd^2+^ and Cu^2+^) and co-existing anions (Cl^−^ and/or NO_3_^−^) from wastewater [43]. Compared to the pristine MXene membrane, the hydroxylated MXene showcased much-enhanced characteristics in terms of hydrophilicity and surface charge, along with enhanced ions separation. With a photocatalytic self-cleaning activity, Lin et al. (2022) synthesized a novel nanocomposite membrane comprising a separation layer of chitosan-coated MXene nanosheets and deposited it on a support layer of a g-C_3_N_4_ photocatalyst-modified PVDF mixed-matrix membrane [44]. Experimental results revealed high water permeability with considerable separation against anionic dyes (>98% for Congo red) and heavy metals (85% for Cr^6+^).

Herein, MXene has been suggested for fabricating a nanocomposite membrane comprising MXene and PPSU for nanofiltration of heavy metal ions removal using a facile, cost-effective and rapid method. So far, in the literature, there is a limited amount of research considering the applications of the novel MXene for polymeric membrane modification compared to conventional nanoscale materials. Additionally, most research focuses on fabricating a standalone MXene membrane (thin layer), while only a few papers reported the application of MXene for mixed-matrix membrane fabrication. In addition, no work has considered using PPSU as a host polymer for MXene mixed-matrix membrane fabrication or MXene-modified NF membranes for heavy metals removal.

## 2. Materials and Methodologies

### 2.1. Materials

Polyphenylsulfone (PPSU) polymer (Radel^®^ R-5000) with a molecular weight of 52,000–55,000 g/mol was purchased from Solvay Advanced Polymer, Belgium. Dimethyl acetamide (DMAC) (85, wt.%, Fisher, Hampton, NH, USA), used as an organic solvent for polymer dissociation, was purchased from the local market. Polyvinylpyrrolidone K30 (PVP-K30) was used as a pore former and purchased from Sigma-Aldrich, St. Louis, MO, USA. MXene (structure; Ti_3_C_2_T_x_) was obtained from the department of materials, University of Technology, Iraq. Cu (NO_3_)_2_·3H_2_O, Cd (NO_3_)_2_·4H_2_O and Pb(NO_3_)_2_ salts for filtration tests of the Cu^2+^, Cd^2+^ and Pb^2+^ heavy metal ions were purchased from Sigma-Aldrich, USA. All chemicals were in analytical grades and used without further purification.

### 2.2. Nanocomposite Membrane Fabrication

The neat and MXene-modified NF membranes have been fabricated via the non-induced phase inversion (NIPS) process. For preparing the membranes, a fixed amount of the PPSU polymer and PVP were dissolved in DMAC with continuous stirring at 50 °C overnight. The required composition of MXene was added to the solution and sonicated for an hour in an ultrasonication bath to achieve a homogenous dispersion and avoid agglomeration of MXene. Following that, the casting solution was left aside to get rid of air bubbles (degassing) and an appropriate amount of the solution was poured on a clean and dry glass substrate which was then cast using an automated casting machine. Finally, the film was directly placed in a tab water bath for coagulation. After the phase inversion process completion, the formed membrane was taken out, rinsed with deionized (DI) water and stored wet in a sealed container, ready for characterization. The composition of all control and modified NF membranes is given in Table 1 below. A code was denoted for each membrane depending on the MXene composition in that membrane.

### 2.3. Nanocomposite Membrane Characterization

To investigate the influence of the MXene content on the PPSU membrane porosity, a 2 cm × 2 cm sample of each membrane was cut precisely and soaked in water. The samples were then wiped with a tissue to remove excessive drops at the surface and the weight is recorded. Following that, the samples were dried naturally at room temperature overnight. The dry weight of the samples was measured, and the porosity of each membrane is calculated according to the following equation [45]:

(1) $$\rho \left(\%\right)=\frac{\left(W_{wet}-W_{dry}\right)/D_{w}}{\left(W_{wet}-W_{dry}\right)/D_{w}+\left(W_{dry}/D_{p}\right)}\times 100\%\;\;\;\;\;\;\;\;\;\;\;\;$$

where  ρ = porosity of membrane (%), *W_wet_* = wet membrane weight (g), *W_dry_* = dry membrane weight (g), *D_w_* = density of water (0.998 g/cm^3^) and *D_p_* = density of PPSU polymer (1.3 g/cm^3^).

The mean pore radius (*r_m_*) of the membranes was determined according to Guerout–Elford–Ferry equation, as given below [45];

(2) $$r_{m\;}=\sqrt{\frac{\left(2.9-1.75\varepsilon \right)\times 8\mu hJ_{w}}{\varepsilon ∆P}}\;\;\;$$

where *ε* = porosity of membrane, *μ* = water viscosity (0.00089 Pa·s), *h* = membrane thickness (m), *J_w_* = water flux (m^3^/m^2^·s) and ∆*P* = differential pressure.

Scanning electron microscopy (SEM) (JEOL, JIB 4700F) has been utilized to visualize the morphology of the surface and cross-section of the membranes before and after modification with various MXene contents. Before SEM imaging, membrane samples were cut into strips and frozen in liquid nitrogen. The membrane strips were then directly fractured and coated with a 5-nm thick gold layer using a sputtering coat machine. The sample was then attached to an appropriate sample holder and placed inside the SEM chamber ready for imaging.

The surface topography of the membrane was scanned using atomic force microscopy (MultiMode 8 AFM) with NanoScope V Controller. The measurements were conducted via a tapping mode in the air at room temperature using a silicon tip and were analyzed using special AFM software to determine the surface roughness parameters, including the root mean square roughness (R_q_) and the average roughness (R_a_).

To confirm the successful incorporation of MXene within the PPSU polymeric membranes, a Fourier-transform infrared spectrophotometry (FT-IR) has been employed. FTIR is a powerful tool for determining the molecular structure and existence of various hydrophilic functional groups. A background spectrum was initially taken as a baseline and then the MXene, PPSU membrane and MXene-modified membrane final spectrum is recorded. All transmittance spectra were taken within a range of 4000 cm^−1^ to 400 cm^−1^.

Hydrophilicity measurements of the membranes were evaluated via the sessile drop method. An optical contact angle instrument (CAM200, KSV instrument Ltd., Helsinki, Finland) connected to PC software was utilized to conduct the membranes’ CA measurement. The membrane sample was cut into 4 × 1 cm strips and attached to a glass microscope slide. Then, a 3 μL DI water drop was placed by the instrument’s syringe on the flattened membrane surface. The contact angle of the drop with the flat membrane surface was captured using the camera and processed using the instrument software.

An Atomic Absorption Spectrophotometer (AAS) instrument (iCE 3000 Series AA Spectrometers) has been harnessed to acquire the element concentration. The concentration of Cu^2+^, Cd^2+^ and Pb^2+^ in the feed and the permeation was measured at a wavelength of 324.8 nm, 217.0 nm and 228.8 nm, respectively.

### 2.4. Performance Evaluation

The water permeation and heavy metal ions retention of all NF membranes were determined using a custom-made crossflow testing rig. The system is composed of a feed tank, circulating pump, flowmeter, two pressure gauges, membrane cell and Teflon tubes. The membrane cell was constructed from Teflon and has an active filtration area of 14.4 cm^2^. More details with schematic diagrams of the testing rig were presented elsewhere [46]. For evaluation and comparing the permeability performance, the NF membranes were initially compacted utilizing DI water for 30 min at 5 bars. The operating pressure was then fixed at 3 bars and the pure water flux of each membrane is measured according to Equation (3) below.

(3) $$\mathrm{Water}\;\mathrm{flux}\;\left(\mathrm{Jw}\right)=\frac{V}{A\cdot ∆t}$$

where Jw: water flux (L/m^2^·h), V: permeated water volume (L), Δt: the measurement period (h) and A: membrane area (m^2^).

Following the pure water flux measurements, the membrane retention performance against three heavy metal ion synthetic solutions was conducted. Two concentrations (10 and 50 ppm) of copper, cadmium and lead-contaminated solutions have been utilized to assess the performance of the membrane. An amount of 250 mL of each concentration (10 and 50 mg/L) of the three heavy metal solutions was passed through the selected membrane at the 3-bar transmembrane pressure. About 20 mL of the permeate samples have been collected at 10-min intervals and the concentration of the heavy metals was measured utilizing Atomic Absorption Spectroscopy (AAS) after calibration. The rejection magnitude was calculated according to the following equation.

(4) $$R\%=1-\frac{C_{p}}{C_{f}}\times 100$$

where R%: is the retention percentage, C_p_: solute concentration in the permeate and C_f_: solute concentration in the feed.

The fouled membrane was then backwashed with DI water for 30 min and the water flux is recorded. The total fouling (F_Total_) of the membrane induced by various heavy metal ions, the reversible fouling (F_r_) and the irreversible fouling (F_irr_) were determined as given below.

(5) $$F_{\mathrm{Total}}=F_{\mathrm{rev}}+F_{\mathrm{irrev}}$$

(6) $$F_{\mathrm{Total}}\left(100\%\right)=1-\frac{J_{1}}{J_{0}}\times 100$$

(7) $$F_{\mathrm{rev}}\;\left(100\%\right)=\frac{J_{2}-{\;J}_{1}}{J_{0}}\times 100$$

(8) $$F_{\mathrm{irrev}}\left(100\%\right)=\frac{J_{0}-{\;J}_{2}}{J_{0}}\times 100$$

where J_0_ = the initial water flux of control membrane, J_1_ = the solute flux of heavy metal ions and J_2_ = the water flux of fouled membrane after cleaning. All measurements are replicates and an average value was presented [47].

## 3. Results and Discussion

### 3.1. MXene-Modified Nanocomposite Membranes

To provide a systematic evaluation of the characteristics and performance of the MXene-modified membranes, a comprehensive characterization was performed to determine the optimal membrane attributes. The membranes were characterized in terms of surface and cross-sectional morphology, composition, surface roughness, hydrophilicity, mean pore and porosity. Likewise, membrane performance was evaluated to determine how efficient these membranes depending on the MXene content used are. This was conducted by measuring and comparing the pure water flux, rejection against three heavy metal ions (Cu, Cd and Pb) and the fouling behavior of these nanocomposite membranes under operation conditions. It should be noted that there was a trivial gradual difference in the color of the membrane’s top surface upon increasing the MXene content in the nanocomposite membranes. This change in the color was proportional as the PPSU control membrane surface was white while tending to turn into darker grey at the 1.5-MXene membrane. However, the bottom sides of all membranes were much lighter than the top surface. This indicates that MXene have migrated during the phase separation process and accumulated close to the surface of the membrane. Practically, this is a well-desired feature that could impart the hydrophilic characteristics of MXene close to the surface of the nanocomposite membrane.

**Figure 1:**
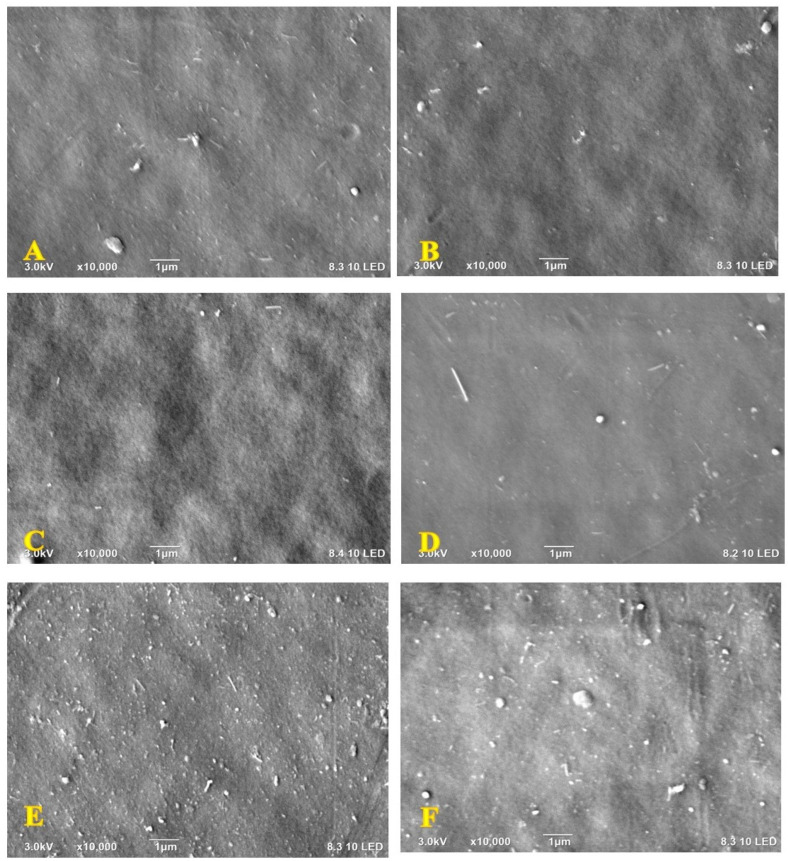
Microscopic images for the surface of (**A**) control PPSU, (**B**) 0.1-M, (**C**) 0.3-M, (**D**) 0.6-M, (**E**) 1-M and (**F**) 1.5-M membrane.

Scanning electron microscopy (SEM) has been employed to visualize the MXene content influence on both surface and cross-sectional morphologies of the fabricated NF nanocomposite membranes. As depicted in Figure 1, a smooth surface was unsurprisingly observed for all samples which are normally associated with sulfone derivative membranes [48]. It is obvious that the MXene displayed good stability due to the efficient dispersion within the PPSU polymeric matrix, and there were no apparent cracks at the surface. No clear MXene aggregates were accumulated at the surface of the NF membranes, even at high MXene loading weights (Figure 1B–F). Despite not being observed, their accumulation at the surface implies that MXene was wrapped with the PPSU polymer close to the surface.

**Figure 2:**
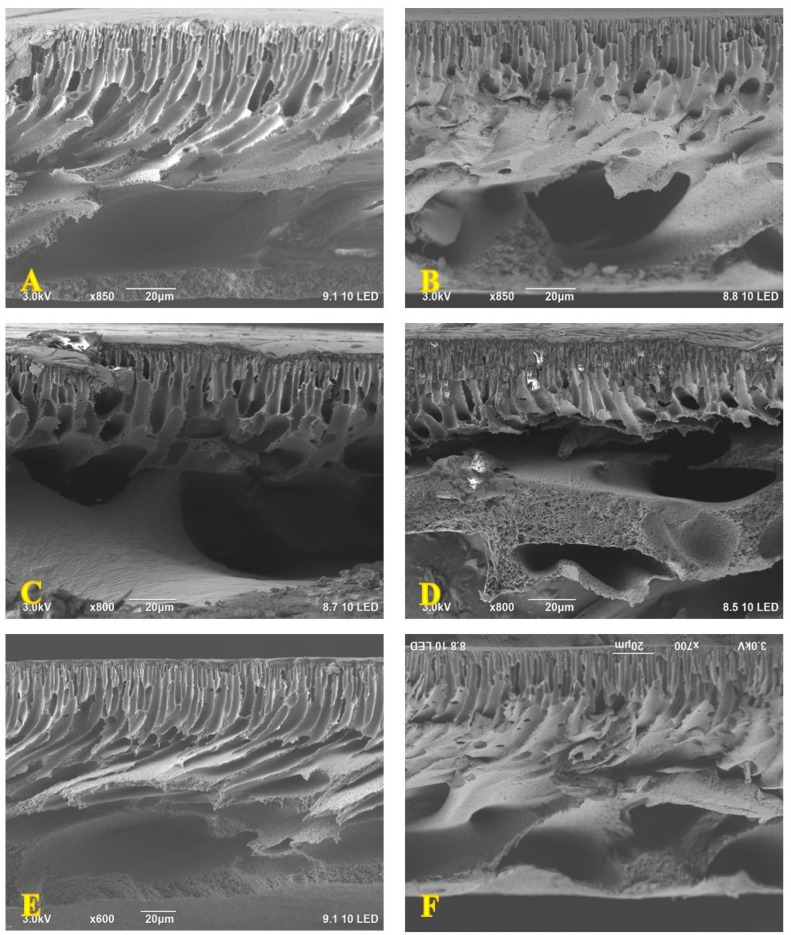
Cross-sectional morphology of (**A**) control PPSU, (**B**) 0.1-M, (**C**) 0.3-M, (**D**) 0.6-M, (**E**) 1-M and (**F**) 1.5-M membrane.

Meanwhile, the impact of 2D MXene nanosheets on the cross-section of the PPSU membrane was more distinguishable, as depicted in Figure 2. Initially, the control membrane sample revealed a common asymmetric structure that comprises a thin, dense top layer supported by a finger-like structure sublayer in the top half section [49]. Likewise, a wide porous structure was observed at the bottom half of the membrane (Figure 2A). However, the incorporation of the nano additives within the polymeric matrix at 0.1 wt.% has slightly reduced the thickness of the figure-like structure (see Figure 2B). This figure-like structure became noticeably thinner at a higher MXene content (0.3 and 0.6 wt.%) and reduced to become about one-third of the cross-section. Additionally, a thinner active layer was observed at the top of the 0.3-M and 0.6-M membranes. These changes could bestow greater permeability features to these membranes, as will be described later in this chapter. As shown in Figure 2, there was no apparent variation in the number of finger-like pores or their size. With a further amount of MXene (1 and 1.5 wt.%), the figure-like structure became thicker again, covered the entire upper half of the cross-section and produced a thicker active layer (Figure 2E,F). Meanwhile, all membranes revealed a much wider structure, showing huge macro voids below the figure-like structure. Herein, no distinguished variation was observed between the membranes. These observations were due to the fixed amount of PPSU polymers from one side and the very low MXene content harnessed to prepare all the NF membranes from the other side. Indeed, there was a trivial variation in the density of the casting solution and ultimately the mixing–de-mixing during the NIPS process. These results are in agreement with the preceding literature [48].

**Figure 3:**
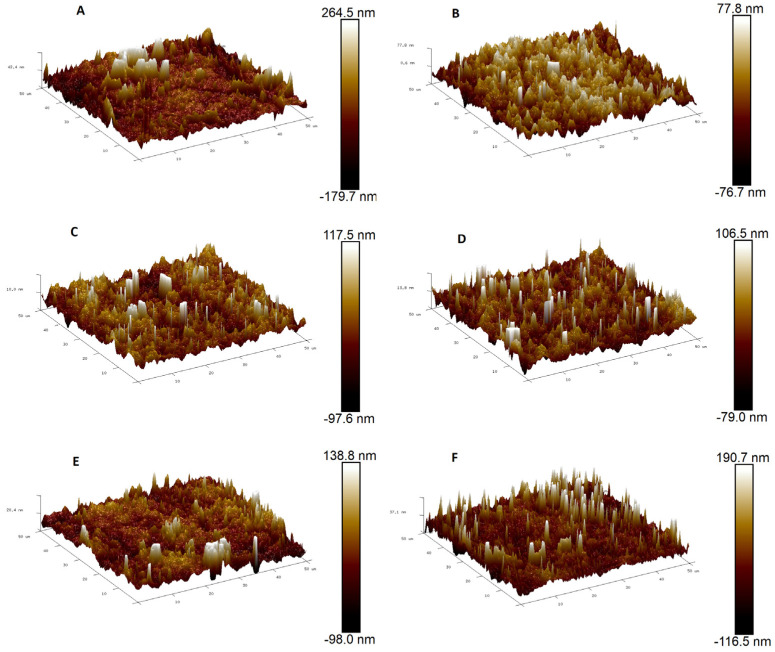
50 μm × 50 μm atomic force microscopy images for (**A**) control PPSU membrane, (**B**) 0.1 wt.%, (**C**) 0.3 wt.%, (**D**) 0.6 wt.%, (**E**) 1 wt.% and (**F**) 1.5 wt.% MXene-modified NF membranes.

Topography parameters of the control and MXene-modified membrane surface were analyzed using atomic force microscopy (AFM). The 50 μm × 50 μm 3D images of the six membrane samples were illustrated in Figure 3 below. Bright regions in the 3D images referred to the highest points (peaks) at the surface while dark regions referred to the lowest points (valleys). Roughness parameters including arithmetic mean height (R_a_) and root-mean-square height (R_q_) of all membranes were given in Table 2. As could be seen in Figure 3A, the control PPSU membrane revealed a rougher surface if compared to all other nanocomposite membranes. The value of the R_a_ and R_q_ recorded 56.75 nm and 89.3 nm, respectively, which were almost double the rest of the samples. These high roughness values agreed with what was reported in the preceding literature for control membranes [50]. Following the MXene addition, a very notable decline was witnessed in the roughness parameters for all nanocomposite membranes (see Figure 3B–F). Noteworthy, no matter how much MXene content has been harnessed to synthesize the nanocomposite membrane, there was no clear correlation between the MXene content and roughness parameters. All the modified membrane samples showcased comparable R_a_ values ranging between 20.5 nm and 27.3 nm (Table 2). Compared to the control membrane, the R_a_ of the 0.1-m nanocomposite membrane has dropped by 63.8% to show 20.5 nm while the R_q_ was 89.3. Raising the MXene content to 0.3, 0.6, 1 and 1.5 wt.% did not disclose any explicit increasing or decreasing trend, and the variation in R_a_ or R_q_ values was trivial. These results suggest that there was no recognizable influence of the MXene content on the surface roughness of the nanocomposite membranes. Additionally, the homogenous dispersion of MXene within the PPSU polymeric matrix was confirmed, even at the highest content. Qiu et al. (2009) reported a similar behavior when using MXene for PSU membrane modification [51].

**Figure 4:**
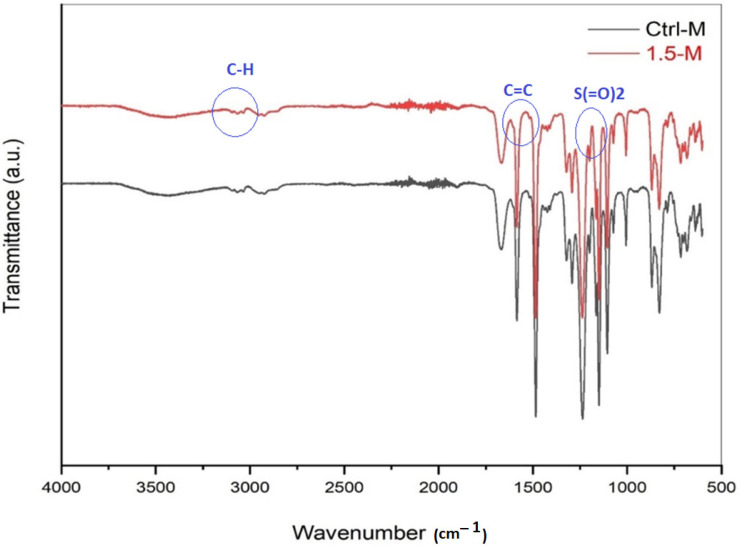
FTIR spectra of control and 1.5 wt.% MXene-modified NF membranes.

The FTIR spectra of neat and 1.5 wt.% MXene-modified nanocomposite membranes were compared, as depicted in Figure 4 below. FTIR analysis is essential to identify the functional groups of the membranes, before and after modification with the MXene nanosheets. Unsurprisingly, both membranes manifested almost the typical spectra of PPSU. The three characteristic peaks that appeared at 3031 cm^−1^, 3066 cm^−1^ and 3092 cm^−1^ are the absorption bands of C–H bonds. The peaks observed at 1482 cm^−1^ and 1584 cm^−1^ are assigned to the C=C bond while the other two spectra at 1237 cm^−1^ and 1322 cm^−1^ are attributed to the S(=O)_2_ bond [40]. In comparison to the control PPSU membrane, no additional peak was visualized following the MXene incorporation in the nanocomposite membrane. Most probably, the MXene bands overlapped with PPSU bands, especially when wrapped with the PPSU polymer, as reported in the surface analysis via SEM earlier. Additionally, the MXene ratio was very low compared to the PPSU, which complicates their detection at the surface. A similar observation was also reported by Kiani et al. (2015) for detecting polyethene glycol in PPSU nanofibrous membranes [52].

**Figure 5:**
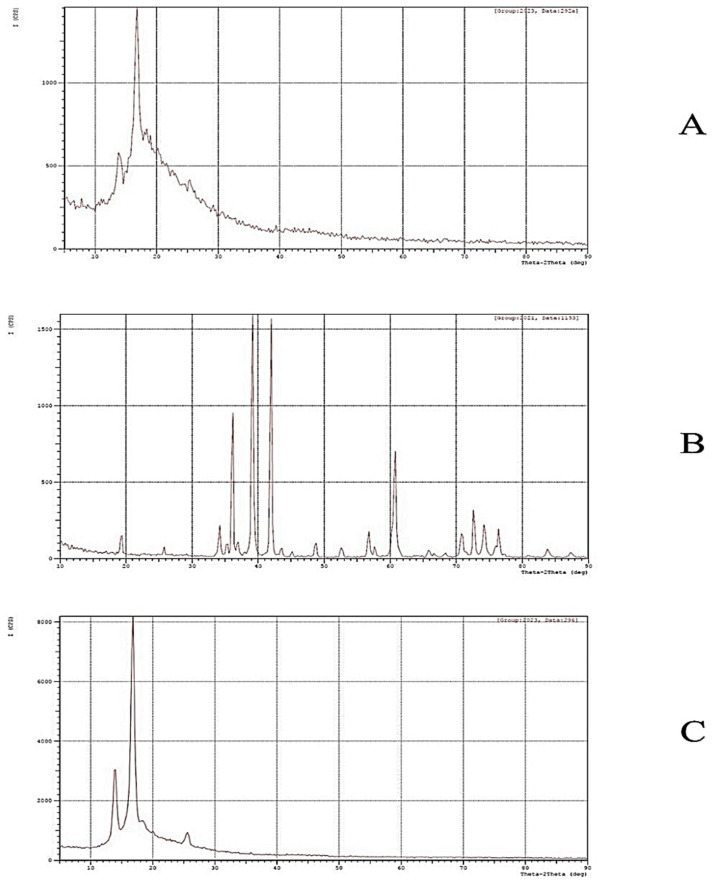
X-ray diffraction of (**A**) PPSU membrane, (**B**) MXene powder and 1.5 wt.% MXene-modified NF membranes (**C**).

The X-ray diffraction patterns of PPSU-, MXene- and MXene-modified PPSU NF membranes are illustrated in Figure 5. The single broad reflection within the range 2θ = 10–35°, seen in Figure 5A, is an indication for the amorphous character of the PPSU diffractogram [53]. The spectrums ascribed to the MXene powder are observed at 2θ = 19.5, 34.3, 36.9, 39.3, 42.0, 45.1, 48.7, 52.4, 56.6 and 60.5°, which corresponded, respectively, to the (004), (101), (103), (104), (105), (106), (107), (108), (109) and (110) crystal planes of Ti_3_AlC_2_ [54] (Figure 5B). Surprisingly, major spectrums of Ti_3_AlC_2_ were not recognizable in the nanocomposite membrane sample (Figure 5C). This could have resulted from the overlapping of the PPSU polymer broad spectra, especially since the MXene percentage harnessed in this work was very trivial compared to the higher rates employed in the other literature.

**Figure 6:**
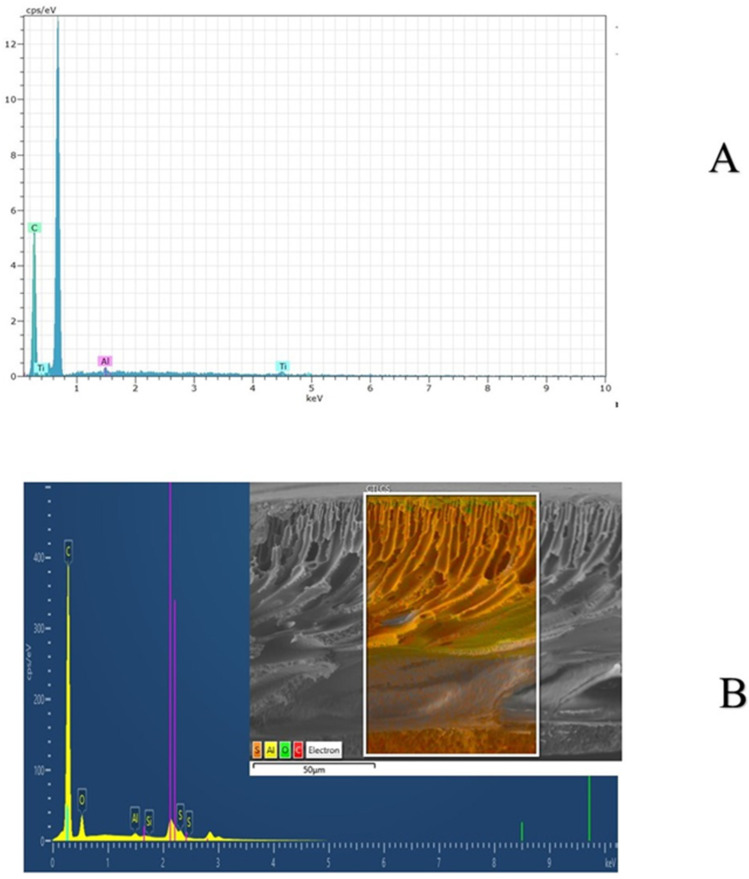
EDX analysis of (**A**) MXene and (**B**) 1.5%MXene-modified PPSU membrane.

Results obtained via the EDX spectrums for the MXene and MXene/PPSU/NF membrane are depicted in Figure 6. The elemental composition of MXene, which is Ti_3_AlC_2_, was confirmed by the presence of Ti, Al and C elements in the spectra at 0.7 and 4.5 keV, 1.5 keV and 0.28 keV, respectively (Figure 6A). Similar to what has been observed in the XRD results, the EDX spectra of the nanocomposite membrane disclosed the domination of the elemental composition of PPSU over the MXene due to the low MXene content adopted. The spectra of PPSU was confirmed by the presence of C, O and S at 0.25 keV, 0.5 keV and 2.28 keV, respectively (Figure 6B).

**Figure 7:**
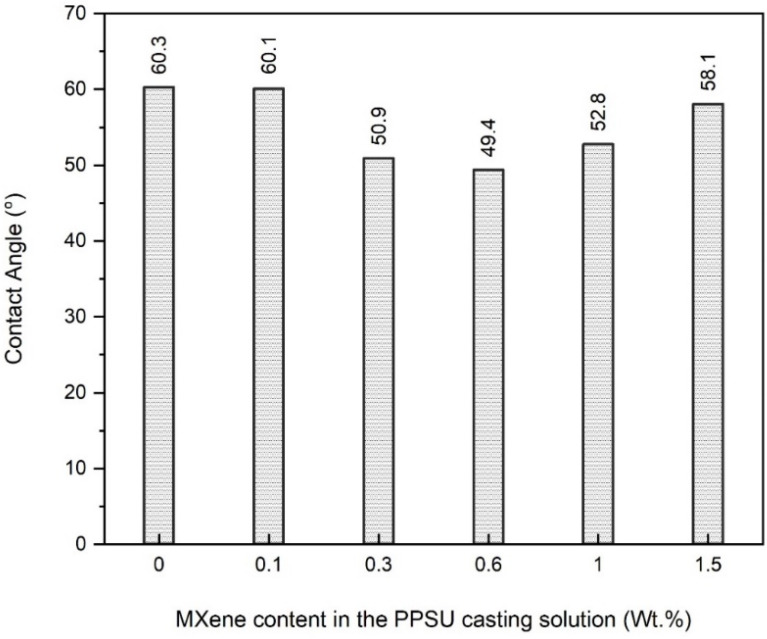
Contact angle measurement of the control and MXene-modified membranes.

The hydrophilicity of a surface is an indication of its affinity to absorb water and is expressed by the contact angle (CA) between the water drop and the surface. A lower contact angle indicates higher hydrophilicity and vice versa. Higher hydrophilicity of the membrane is a well-desirable characteristic for enhancing the permeation, retention and antifouling performance of that membrane. The CA values of the control and MXene-modified membranes are illustrated in Figure 7. The control membrane showed greater CA (60.3°) among all other modified nanocomposite membranes. Adding 0.1 wt.% MXene in the casting solution has given rise to a slightly lower CA (60.1°). Raising the content of MXene to 0.3 and 0.6 wt.% resulted in a significant drop in the CA value and recorded 50.9° and 49.5°, respectively. This is a confirmation that hydrophilic MXene has offered the membrane surface better hydrophilic features. Consequently, oxygen-containing functional groups of MXene are attached to the membrane surface and are expected to endow better antifouling performance [55]. Furthermore, the MXene was homogenously distributed within the membrane structure, as illustrated by the SEM imaging. On the other hand, when the MXene wt.% exceeded the 0.6 limits, the hydrophilicity of the nanocomposite membranes witnessed a gradual decrease to 52.8° and 58.1° for the 1-M and 1.5-M membranes, respectively. This drop in the hydrophilicity was probably attributed to the agglomeration of MXene at the higher loading content where big aggregates were formed. Agglomeration can minimize the surface area of MXene and ultimately, the presence of less hydrophilic functional groups at the membrane surface. This work reported much better hydrophilicity than that reported using MXene for membrane modification [48].

**Figure 8:**
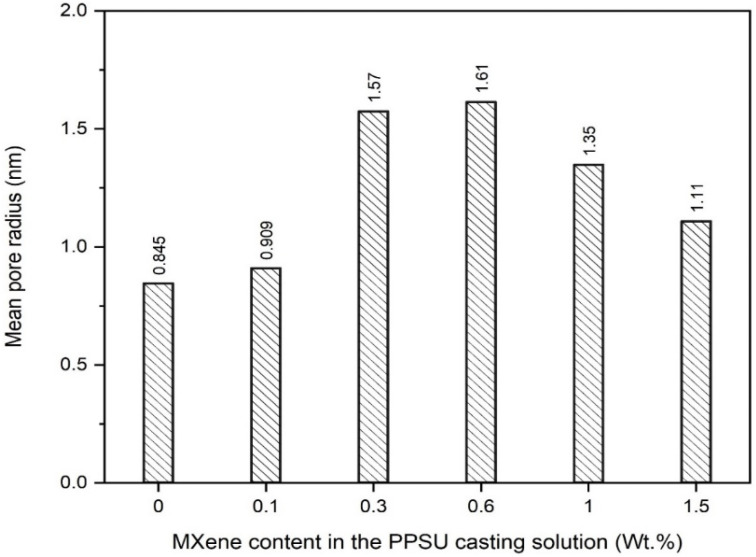
Influence of MXene content on the mean pore size of the NF nanocomposite membranes.

The influence of the MXene content on the mean pore size and porosity of the NF/PPSU nanocomposite membranes was investigated. Pore size and porosity are critical surface characteristics controlling the permeability and retention of any membrane. Smaller pore sizes give rise to a higher rejection rate, while a higher porosity results in higher permeability. Figure 8 showcases the impact of the MXene content incorporated within the polymeric membrane on the mean pore size value. As could be seen, the control PPSU membrane exhibited the lowest mean pore radius (0.8 nm) among all other modified NF nanocomposite membranes. Adding only 0.1 wt.% MXene into the PPSU casting solution (0.1 M) has induced a slight increase in the pore size (~0.9 nm). Increasing the MXene content in the polymeric matrix has shown a further increment in the mean pore size value (~1.5 nm), as seen for the 0.3-M NF membrane, while the highest recorded mean pore radius (1.6 nm) was obtained at 0.6 wt.% MXene. However, the mean pore size upon MXene content addition has shown a slight decrease, where ~1.3 nm and 1.1 nm were observed at an MXene loading ratio of 1 and 1.5 wt.%, respectively. This increase and then decrease in the mean pore size values could be attributed to the presence of hydrophilic MXene in the casting solution, enhancing the mixing–de-mixing process between the solvent (DMAC) and nonsolvent (DI water) during the phase inversion. A similar trend was reported by Shen et al. (2019) for the MXene-modified PES membrane [36]. This enhancement showcased a continuous increasing behavior up to 0.6 wt.% MXene (lowest CA value), where beyond this value a slight drop in the hydrophilicity (higher CA value) was observed, as confirmed using the CA measurements (Figure 7).

**Figure 9:**
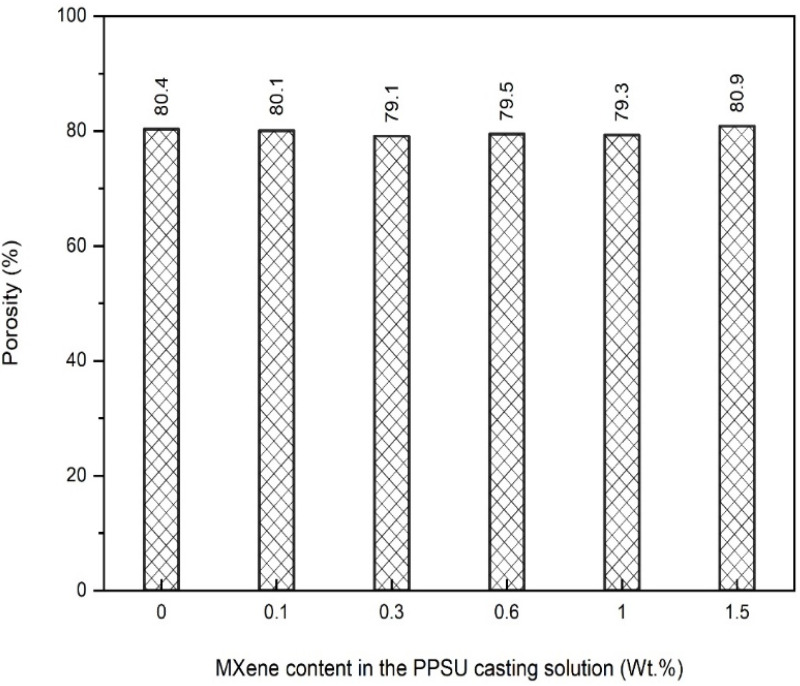
Porosity measurements of the NF nanocomposite membranes.

In the meantime, all control and nanocomposite membranes revealed no change in their porosity values upon varying the MXene content in the casting solution. All fabricated membranes exhibited an average porosity of around 80%, regardless of the MXene wt.% (see Figure 9). This indicates that MXene incorporation at the utilized low-weight precents does not disclose any apparent influence on the porosity value of the membranes. Similar behavior has been seen in preceding research using other nanomaterials [48].

### 3.2. Performance Evaluation of MXene-Modified Nanocomposite Membranes

#### 3.2.1. Pure Water Flux (PWF) of the Nanocomposite Membranes

**Figure 10:**
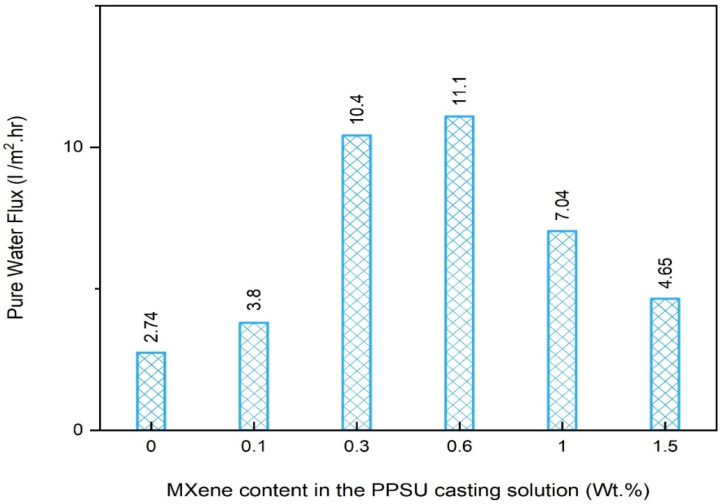
Pure water flux of membranes at various loading weights of MXene and 3-bar operating pressure.

The pure water flux (PWF) of the MXene-modified membranes was measured and compared with that of the control PPSU membrane to investigate the influence of the MXene content on the nanocomposite membrane’s permeability. As illustrated in Figure 10, the control PPSU membrane manifested a minimal pure water flux rate (2.74 LMH) compared to all other MXene-modified membranes. Upon MXene incorporation, the PWF witnessed a 28% improvement and recorded 3.8 LMH when only 0.1 wt.% MXene was used. On the other hand, a surge enhancement in the PWF was seen when raising the MXene content in the casting solution to 0.3 wt.%. The 0.3 m nanocomposite membrane manifested about a four-fold flux (10.4 LMH) compared to that of the control membrane. The uppermost PWF was revealed by the nanocomposite membrane prepared with 0.6 wt.% MXene, and was recorded as 11.1 LMH. This enhancement is mainly attributed to the synergy of both improvements in the hydrophilicity and mean pore size. However, a further amount of MXene (1 wt.%) induced a considerable decline in the water flux value to about 7.04 LMH. Likewise, the highest employed MXene content (1.5 M) revealed the MOST minimal PWF value (4.65 LMH); however, this was still double that of the pristine PPSU membrane. This decline in the permeability of the later nanocomposite membranes probably resulted from the diminished mean pore size and hydrophilicity obtained at those MXene loading ratios. Despite that, results disclosed that at all MXene ratios, the nanocomposite exhibited a superior pure water flux compared to the unmodified PPSU membrane.

#### 3.2.2. Potential Separation of Nanocomposite Membranes

**Figure 11:**
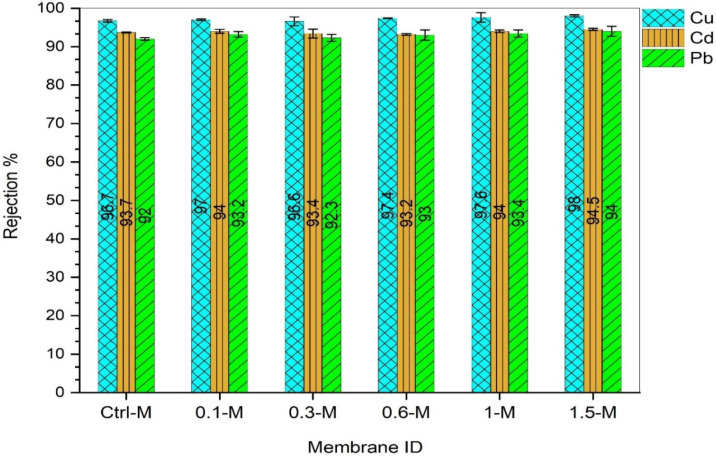
Rejection rate of NF nanocomposite membranes against copper, cadmium and lead feed solutions (50 ppm, 3 bars, pH = 7, 1LMH and 25 °C).

To investigate the influence of the MXene content on the retention potential of the nanocomposite membranes, a lab-scale crossflow filtration apparatus has been employed. All membrane samples were tested against three individual heavy metal ions (copper, cadmium and lead) containing synthetic feed solutions. Experimental conditions were fixed during all measurements where feed concentration = 50 ppm, operating pressure = 3 bars, flowrate = 1 LMH, pH = 7 and temperature = 25 °C. The rejection measurements of the NF membranes against the copper, cadmium and lead ions are compared, as illustrated in Figure 11 below. All control and MXene-modified membranes manifested notably high (92–98%) retention capabilities against all metal ions. Likewise, there was no clear correlation between the separation performance and MXene ratio in the nanocomposite membranes. The order of metal retention efficiency was Cu^2+^ > Cd^2+^ > Pb^2+^, regardless of the membrane type. Compared to other metal ions, the slightly higher rejection of copper could stem from the precipitation of metal hydroxide at an alkaline or neutral pH [56]. The control PPSU membrane, which has the lowest pore size, was able to retain 96.7% of copper ions, while the 0.6-M nanocomposite showed slightly higher retention (97.4%) despite having a bigger pore size. This could be due to several reasons, including the rapid precipitation of copper hydroxide at the membrane surface forming a cake layer. Additionally, the membrane rejection was dependent not only on pore size but also on surface charge [57]. Most probably, an additional surface charge was imparted on the nanocomposite membranes due to the presence of MXene functional groups. The surface charge effect was more obvious in the Cd^2+^ ions’ retention, which exhibited lower values than Cu^2+^, despite Cu^2+^ having a lower hydrodynamic radius. The variation in the Cd^2+^ retention between the control and all nanocomposite membranes was trivial and ranged between 93.2% and 94.5%. Nonetheless, the retention of Pb^2+^ ions was slightly lower than the Cd^2+^ ions and recorded separation rates between 92–94%. These results enclosed that all control and modified membranes have comparable separation potential, despite a significant variation in their permeabilities and pore size.

#### 3.2.3. Fouling Behavior of Nanocomposite Membranes

**Figure 12:**
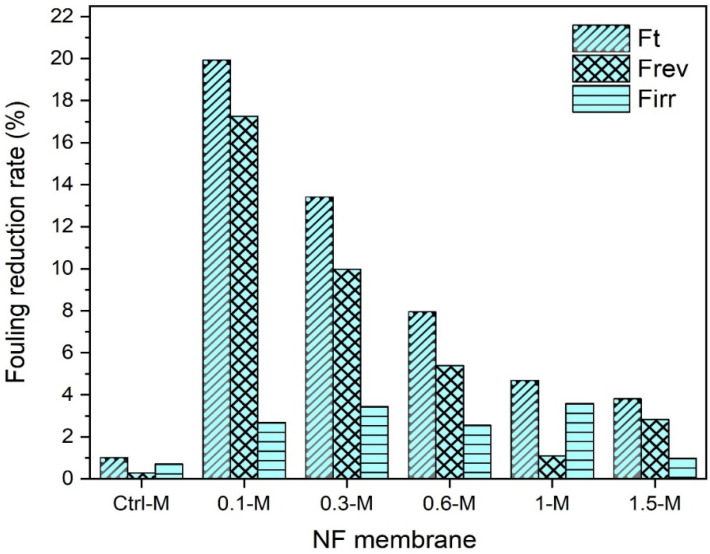
Fouling behavior of NF membranes with 50-ppm copper solution.

Fouling is the major cause of membrane flux decline that diminishes membrane performance. Depending on the membrane surface characteristics, operating conditions and solute nature, disparate fouling scenarios could be seen, such as internal pore blocking and narrowing, deposition and cake formation [58]. To evaluate the influence of MXene on the membrane performance, the total reversible and irreversible fouling of the membranes was determined against the heavy metals synthetic solutions. For each membrane, the test was initiated by testing the pure water flux and followed by measuring the flux of the solute (heavy metal ions solution), and the flux decline was then recorded. The membrane was then backwashed for 30 min, and the pure water flux was measured again. The fouling behavior of the control and modified nanocomposite membranes against copper ions solution were illustrated in Figure 12. Although the control PPSU membrane manifested the lowest (~1%) total reduction fouling rate due to the smallest pore size obtained, most of that rate was irreversible. In contrast, incorporating only 0.1 wt.% MXene in the polymeric matrix showcased the highest total reduction fouling rate (19.9%) compared to other nanocomposites prepared with a higher MXene content. As could be seen, the total reduction fouling rates against copper feed solution were indirectly proportional to the MXene ratio in the nanocomposite membrane. The nanocomposite membranes recorded 13.4, 7.9, 4.68 and 3.8% total fouling for 0.3-M, 0.6-M, 1-M and 1.5-M, respectively. Despite the nanocomposite membranes revealing higher reduction fouling rates, the flux decline was mostly recoverable and only a minor fouling rate was irreversible. This indicated that Cu ions were loosely attached to the surface of the nanocomposite membranes and were easily washable. This was probably induced by the smoother and more hydrophilic surface of nanocomposite membranes compared to the control membrane. It is worth mentioning that the final flux of all nanocomposites after backwashing was much higher than that of the control membrane and comparable to their original values.

**Figure 13:**
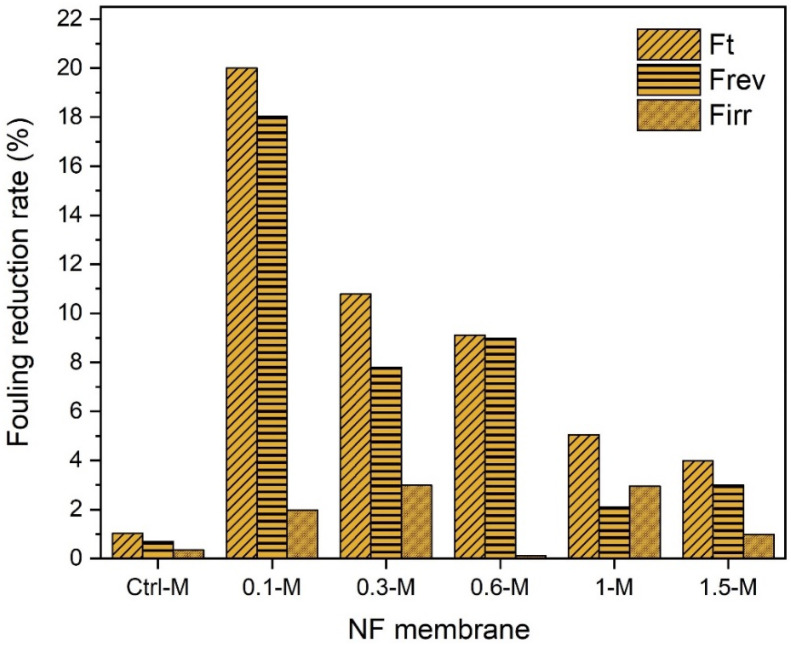
Fouling behavior of NF membranes with 50-ppm cadmium solution.

**Figure 14:**
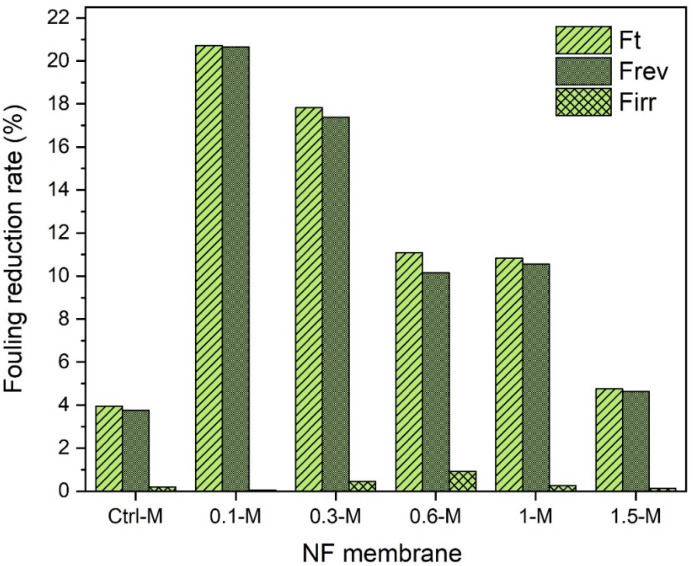
Fouling behavior of NF membranes with 50-ppm lead solution.

A similar performance trend was also observed for the cadmium feed solutions, as shown in Figure 13. The total reduction in the fouling of the control membrane was 1%, while 20, 10.79, 9.1, 5 and 3.9% was recorded for the 0.1-M, 0.3-M, 0.6-M, 1-M and 1.5-M nanocomposites, respectively. The same high reversible fouling rates experienced with copper ions were also witnessed with the cadmium solution. This recognizable reversible fouling was more obvious when filtrating the lead solution (see Figure 12). Even though all membranes exhibited slightly higher reduction fouling rates against the lead solution, almost a complete flux could be restored by 30 min of backwashing. At neutral pH conditions, Pb^2+^ could be easily fixed by membrane surface charges, and this could disturb the solute passage through the membrane due to reduced pore sizes [59]. The control membrane manifested around a ~4% total reduction in the fouling rate when filtrating the lead solution as shown in Figure 14 compared to only ~1% in the case of copper and cadmium. The order of metals causing a higher reduction fouling rate was Pb^2+^ > Cd^2+^ > Cu^2+^. These results disagreed with several other reported studies that ascribed higher fouling to be associated with copper ions [60], while others ascribed it to cadmium ions [61].

To explain the results, the permeate flux reduction could occur due to many reasons, such as the concentration polarization, adsorption of solutes on the membrane surface, cake layer formation due to metal hydroxide precipitate and osmotic pressure [61].

#### 3.2.4. Comparison Study

Table 3 depicts a comparison study between the performance of PPSU/MXene nanosheets membranes prepared via the current study and the performance of prepared membranes from different polymers and additives and commercial membranes presented in the literature. It can be seen that the PPSU/MXene nanosheets membranes have good and reasonable PWP and heavy metals removal efficiency in comparison with most membranes presented in the literature. Ultimately, these nanocomposites membranes (PPSU/MXene nanosheets) could be an excellent choice for the environmental and economic feasibility of wastewater treatment compared to the preceding literature, as shown in Table 3.

## 4. Limitations of MXene Modified Membranes

Despite the relatively reported promising potential of MXene in membrane field applications, there is still a long way to go before a comprehensive evaluation can be performed. Similar to all metal-based nanoscale materials, MXene should undergo thorough extensive investigation to evaluate its stability inside the polymeric membrane and final destination into water streams. In this context, a pilot plant and long-term experiment are essential to assess the real service life of the membrane. On the other hand, there are a number of reports that claimed MXene poses trivial toxicity, while others reported high toxicity to stem cells [69]. However, this controversy about toxicity is generally associated with the material’s dose, mode of exposure, cell type and specific MXene types. It has also been reported that MXene stability is a major concern, as degradation of MXene may produce toxic byproducts with potential impacts on the ecosystem [70]. Going further, MXene is prone to oxidation, and it is unclear where this issue would limit the MXene prospects [71].

## 5. Conclusions

This study presented an endeavor to fabricate a novel nanocomposite NF membrane that comprises polyphenylsulfone (PPSU) polymer and 2D MXene nanosheets for heavy metals removal from wastewater. Results disclosed that playing with the MXene content could significantly influence some of the surface characteristics of the membranes, whereas no effect was seen on others. The roughness of the nanocomposite membranes was notably enhanced by obtaining a ~50% smoother surface compared to the control PPSU membrane (56.75 nm). In addition, the wettability of the nanocomposite membrane showcased a gradual improvement and reached its maximum value at a 0.6 wt.% MXene ratio (contact angle = 49.4°) compared to only 60.3° for the control membrane. Similarly, the mean pore size was doubled from 0.84 nm to 1.6 for the control and 0.6 wt.% MXene-modified membrane, respectively, while no observable change in the porosity was obtained for all membranes (around 79%).

Alongside that, performance evaluations demonstrated that upon MXene incorporation, the pure water flux witnessed a 28% improvement and recorded 3.8 LMH when only 0.1 wt.% MXene was used. The uppermost flux (11.1 LMH) was revealed by the nanocomposite membrane prepared with 0.6 wt.% MXene, compared to only 2.74 LMH for the control PPSU membrane. Likewise, there was a trivial non palpable change in the retention potentials of all control and modified membranes against all metal ions solutions. In this context, the retention was higher (96–98%) for Cu^2+^ compared to Cd^2+^ and Pd^2+^, which exhibited slightly lower rejections of 93.2–94.5% and 92–94%, respectively. In the meantime, the nanocomposite membranes manifested distinguished antifouling features and were proportional to the MXene content in the nanocomposite membrane. Even though the total reduction fouling reached up to 20% for the 0.1 wt.% MXene-modified membrane, the flux was mostly recoverable and still much higher than the control PPSU membrane.

Obtained results disclosed that MXene-functionalized membranes could provide superior potential for the functional nanofiltration of heavy metals contaminated wastewater effluents.

## Figures and Tables

**Table 1:** Composition of NF membranes.

Membrane ID	PPSU wt.%	PVP wt.%	DMAC wt.%	MXene (g)
Ctrl-M	20	2	78	0
0.1-M	20	2	78	0.1
0.3-M	20	2	78	0.3
0.6-M	20	2	78	0.6
1-M	20	2	78	1
1.5-M	20	2	78	1.5

**Table 2:** Roughness parameters over a 50 × 50 µm scan size.

Membrane ID	R_a_	R_q_
Ctrl-M	56.75	89.3
0.1-M	20.52	30.85
0.3-M	23.4	36.03
0.6-M	20.95	32
1.0-M	27.32	44.64
1.5-M	23.9	37.13

**Table 3:** Comparison of preceding studies with current work in terms of membrane performance for heavy metals removal.

Type of Modification	Application	Flux (LMH)	Rejection (%)	Ref.
PVDF/APTES functionalized halloysite-Magnetic graphene oxide/metformin	Aqueous solution Cu^2+^ Cd^2+^ Cr^2+^	14.2	Cu^2+^ = 47.9% Cd^2+^ = 44.2% Cr^2+^ = 52.3%	[62]
Dual layer polybenzimidazole/PES	Aqueous solution Cr^2+^ Pb^2+^ Cd^2+^	8.3	Cr^2+^ = 98% Pb^2+^ = 93% Cd^2+^ = 70%	[63]
NF270	Pb(NO_3_)_2_/Cd(NO_3_)_2_ aqueous solution	-	Cd^2+^ = 99% Pb^2+^ = 74%	[60]
(PEI) cross-linked P84	Pb(NO_3_)_2_ aqueous solution	-	Pb^2+^ = 91.05%	[64]
TFC-NF300 polyamide thin film	CdCl_2_; NiSO_4_ aqueous solution	-	Cd^2+^ = 80% Ni^2+^ = 97%	[65]
cellulose acetate (CA) NF-23	Cd(NO_3_)_2_	-	Cd^2+^ = 84%	[66]
PES hollow fiber	Ternary aqueous solution Pb^2+^ Cd^2+^ Co^2+^	16.4 37.9 16.6	Pb^2+^ = 40% Cd^2+^ = 48.3% Co^2+^ = 50.5%	[67]
PES hollow fiber	Binary aqueous solution Pb^2+^ Co^2+^ Cd^2+^	-	Pb^2+^ = 60.3% Co^2+^ = 58% Cd^2+^ = 44.5%	[68]
**PPSU NF** bulk modification MXene-modified	Wastewater	11.1	97% for copper, 93.4% for cadmium, and 93% for lead	**This work**

## Data Availability

Not applicable.

## Nomenclature

2D	two dimensions
PPSU	polyphenyl sulfone
AFM	stomic force microscopy
SEM	scanning electron microscopy
FTIR	Fourier-transform infrared spectroscopy
Wt.%	weight percentage
Cu^2+^	copper ions
Cd^2+^	cadmium ions
Pd^2+^	lead ions
0D	zero dimension
1D	one dimension
PVDF	polyvinylidene fluoride
TiO_2_	titanium dioxide
O	oxygen
OH	hydroxyl
F	fluorine
MgCl_2_	magnesium chloride
NaCl	sodium chloride
Cl^−^	chloride ion
NO_3_^−^	nitrates ions
g-C_3_N_4_	graphitic Carbon Nitride
Cr^6+^	chromium ions
NF	nanofiltration
DMAC	dimethyl acetamide
PVP	polyvinylpyrrolidone
Ti_3_C_2_T_x_	MXene
Cu (NO_3_)_2_·3H_2_O	copper nitrate trihydrate
Cd (NO_3_)_2_·4H_2_O	cadmium nitrate tetrahydrate
Pb(NO_3_)_2_	lead (II) nitrate
DI	deionized
ρ	porosity of membrane (%)
W_wet_	wet membrane weight (g)
W_dry_	dry membrane weight (g)
D_w_	density of water (0.998 g/cm^3^)
D_p_	density of PPSU polymer (1.3 g/cm^3^)
r_m_	mean pore radius
μ	water viscosity (0.00089 Pa·s)
h	membrane thickness (m)
J_w_	water flux (m^3^/m^2^·s)
∆P	differential pressure
R_q_	root mean square roughness
R_a_	the average roughness
Jw	water flux (L/m^2^·h)
V	permeated water volume (L),
Δt	the measurement period (h),
A	membrane area (m^2^).
R%:	retention percentage
C_p_	solute concentration in the permeate
C_f_	solute concentration in the feed.
J_0_	initial water flux of control membrane
J_1_	solute flux of heavy metal ions
J_2_	water flux of fouled membrane after cleaning
CA	Contact angle
PES	Polyethersulfone
PWF	Pure water flux

## References

[1] Sobhanardakani S., Tayebi L., Farmany A. (2011). Toxic metal (Pb, Hg and As) contamination of muscle, gill and liver tissues of Otolithes rubber, Pampus argenteus, Parastromateus niger, Scomberomorus commerson and Onchorynchus mykiss. World Appl. Sci. J..

[2] Barakat M. (2011). New trends in removing heavy metals from industrial wastewater. Arab. J. Chem..

[3] Sun Y., Zhou S., Pan S.-Y., Zhu S., Yu Y., Zheng H. (2020). Performance evaluation and optimization of flocculation process for removing heavy metal. Chem. Eng. J..

[4] Burakov A.E., Galunin E.V., Burakova I.V., Kucherova A.E., Agarwal S., Tkachev A.G., Gupta V.K. (2018). Adsorption of heavy metals on conventional and nanostructured materials for wastewater treatment purposes: A review. Ecotoxicol. Environ. Saf..

[5] Matlock M.M., Howerton B.S., Atwood D.A. (2002). Chemical precipitation of heavy metals from acid mine drainage. Water Res..

[6] Bashir A., Malik L.A., Ahad S., Manzoor T., Bhat M.A., Dar G., Pandith A.H. (2019). Removal of heavy metal ions from aqueous system by ion-exchange and biosorption methods. Environ. Chem. Lett..

[7] Shrestha R., Ban S., Devkota S., Sharma S., Joshi R., Tiwari A.P., Kim H.Y., Joshi M.K. (2021). Technological trends in heavy metals removal from industrial wastewater: A review. J. Environ. Chem. Eng..

[8] Awual M.R. (2019). Efficient phosphate removal from water for controlling eutrophication using novel composite adsorbent. J. Clean. Prod..

[9] Perumal S., Atchudan R., Edison T.N.J.I., Babu R.S., Karpagavinayagam P., Vedhi C. (2021). A Short Review on Recent Advances of Hydrogel-Based Adsorbents for Heavy Metal Ions. Metals.

[10] Dièye E.H., Fall A., Fall M., Ferreira C.A., Silveira M.R., Baldissera A.F. (2021). Removal of heavy metals by electrodialysis using polyanilines prepared in hydrochloric acid and ionic liquids. Emergent Mater..

[11] Torma C.Z., Cséfalvay E. (2018). Nanofiltration and electrodialysis: Alternatives in heavy metal containing high salinity process water treatment. Chem. Pap..

[12] Xiao Y., Tan S., Wang D., Wu J., Jia T., Liu Q., Qi Y., Qi X., He P., Zhou M. (2020). CeO_2_/BiOIO_3_ heterojunction with oxygen vacancies and Ce^4+^/Ce^3+^ redox centers synergistically enhanced photocatalytic removal heavy metal. Appl. Surf. Sci..

[13] Zhang Y., Zhang S., Gao J., Chung T.-S. (2016). Layer-by-layer construction of graphene oxide (GO) framework composite membranes for highly efficient heavy metal removal. J. Membr. Sci..

[14] Huang B.-Q., Tang Y.-J., Gao A.-R., Zeng Z.-X., Xue S.-M., Ji C.-H., Tang C.Y., Xu Z.-L. (2021). Dually charged polyamide nanofiltration membranes fabricated by microwave-assisted grafting for heavy metals removal. J. Membr. Sci..

[15] Fu F., Wang Q. (2011). Removal of heavy metal ions from wastewaters: A review. J. Environ. Manag..

[16] Shukla A.K., Alam J., Alhoshan M., Dass L.A., Muthumareeswaran M. (2017). Development of a nanocomposite ultrafiltration membrane based on polyphenylsulfone blended with graphene oxide. Sci. Rep..

[17] Kango S., Kalia S., Celli A., Njuguna J., Habibi Y., Kumar R. (2013). Surface modification of inorganic nanoparticles for development of organic–inorganic nanocomposites—A review. Prog. Polym. Sci..

[18] Alsalhy Q.F., Al-Ani F.H., Al-Najar A.E. (2018). A new Sponge-GAC-Sponge membrane module for submerged membrane bioreactor use in hospital wastewater treatment. Biochem. Eng. J..

[19] Alsalhy Q., Merza A., Rashid K., Adam A., Figoli A., Simone S., Drioli E. (2013). Preparation and Characterization of poly(vinyl chloride)/poly (styrene)/poly (ethylene glycol) hollow-fiber membranes. J. Appl. Polym. Sci..

[20] Khayet M., García-Payo M.C., Qusay F.A., Khulbe K.C., Feng C.Y., Matsuura T. (2008). Effects of gas gap type on structural morphology and performance of hollow fibers. J. Membr. Sci..

[21] Jalal Sadiq A., Shabeeb K.M., Khalil B.I., Alsalhy Q.F. (2020). Effect of embedding MWCNT-g-GO with PVC on the performance of PVC membranes for oily wastewater treatment. Chem. Eng. Comun..

[22] Al-Ani D.M., Al-Ani F.H., Alsalhy Q.F., Ibrahim S.S. (2021). Preparation and characterization of nanofiltration membranes from PPSU-PES polymer blend for dyes removal. Chem. Eng. Comun..

[23] Al-Ani F.H., Alsalhy Q.F., Raheem R.S., Rashid K.T., Figoli A. (2020). Experimental Investigation of the Effect of Implanting TiO_2_-NPs on PVC for Long-Term UF Membrane Performance to Treat Refinery Wastewater. Membranes.

[24] Ghadhban M.Y., Majdi H.S., Rashid K.T., Alsalhy Q.F., Lakshmi D.S., Salih I.K., Figoli A. (2020). Removal of Dye from a Leather Tanning Factory by Flat-Sheet Blend Ultrafiltration (UF) Membrane. Membranes.

[25] Sadiq A.J., Awad E.S., Shabeeb K.M., Khalil B.I., Al-Jubouri S.M., Sabirova T.M., Tretyakova N.A., Majdi H.S., Alsalhy Q.F., Braihi A.J. (2023). Comparative study of embedded functionalised MWCNTs and GO in Ultrafiltration (UF) PVC membrane: Interaction mechanisms and performance. Int. J. Environ. Anal. Chem..

[26] Kadhim R.J., Al-Ani F.H., Alsalhy Q.F. (2021). MCM-41 mesoporous modified polyethersulfone nanofiltration membranes and their prospects fordyes removal. Int. J. Environ. Anal. Chem..

[27] Yahya A.A., Rashid K.T., Ghadhban M.Y., Mousa N.E., Majdi H.S., Salih I.K., Alsalhy Q.F. (2021). Removal of 4-Nitrophenol from Aqueous Solution by Using Polyphenylsulfone-Based Blend Membranes: Characterization and Performance. Membranes.

[28] Cardinale A.M., Carbone C., Fortunato M., Fabiano B., Reverberi A.P. (2022). ZnAl-SO_4_ Layered Double Hydroxide and Allophane for Cr (VI), Cu (II) and Fe (III) Adsorption in Wastewater: Structure Comparison and Synergistic Effects. Materials.

[29] Singh S., Kumar V., Anil A.G., Kapoor D., Khasnabis S., Shekar S., Pavithra N., Samuel J., Subramanian S., Singh J. (2021). Adsorption and detoxification of pharmaceutical compounds from wastewater using nanomaterials: A review on mechanism, kinetics, valorization and circular economy. J. Environ. Manag..

[30] Moradi R., Monfared S.M., Amini Y., Dastbaz A. (2016). Vacuum enhanced membrane distillation for trace contaminant removal of heavy metals from water by electrospun PVDF/TiO 2 hybrid membranes. Korean J. Chem. Eng..

[31] Pandey M., Deshmukh K., Pasha S.K.K., Sadasivuni K.K., Deshmukh K., Pasha S.K.K., Kovářík T. (2022). Chapter 10—MXene-based materials for lithium–sulfur and multivalent rechargeable batteries. Mxenes and Their Composites.

[32] Naguib M., Kurtoglu M., Presser V., Lu J., Niu J., Heon M., Hultman L., Gogotsi Y., Barsoum M.W. (2011). Two-dimensional nanocrystals produced by exfoliation of Ti_3_AlC_2_. Adv. Mater..

[33] Naguib M., Mochalin V.N., Barsoum M.W., Gogotsi Y. (2014). 25th anniversary article: MXenes: A new family of two-dimensional materials. Adv. Mater..

[34] Anasori B., Lukatskaya M., Gogotsi Y. (2017). 2D metal carbides and nitrides (MXenes) for energy storage. Nat. Rev. Mater..

[35] Sun Y., Dall’Agnese C., Zhang C., Yang L., Jin X., Dall’Agnese Y., Wang X.-F., Sadasivuni K.K., Deshmukh K., Pasha S.K.K., Kovářík T. (2022). Chapter 14—Applications of MXenes and their composites in catalysis and photoelectrocatalysis. Mxenes and Their Composites.

[36] Vankayala R., Thangudu S., Kuthala N., Kalluru P., Sadasivuni K.K., Deshmukh K., Pasha S.K.K., Kovářík T. (2022). Chapter 15—MXenes and their composites for medical and biomedical applications. Mxenes and Their Composites.

[37] Liu A., Liang X., Ma T., Sadasivuni K.K., Deshmukh K., Pasha S.K.K., Kovářík T. (2022). Chapter 9—MXenes and their composites for lithium- and sodium-ion battery applications. Mxenes and Their Composites.

[38] Saraswathi K., Reddy M.S.B., Rao K.V., Deshmukh K., Ponnamma D., Waseem S., Aly Rakha M.F.I., Pasha S.K.K., Sadasivuni K.K., Sadasivuni K.K., Deshmukh K., Pasha S.K.K., Kovářík T. (2022). Chapter 21—MXenes and their composites for energy harvesting applications. Mxenes and Their Composites.

[39] Lam S.-M., Jaffari Z.H., Yong Z.-J., Sin J.-C., Zeng H., Lin H., Li H., Mohamed A.R., Sadasivuni K.K., Deshmukh K., Pasha S.K.K., Kovářík T. (2022). Chapter 16—MXenes and their composites for potential antimicrobial applications. Mxenes and Their Composites.

[40] Nirogi A., Elsa G., Vijayakumar M., Sankar A.B., Karthik M., Sadasivuni K.K., Deshmukh K., Pasha S.K.K., Kovářík T. (2022). Chapter 11—MXenes and their composites for supercapacitors and hybrid capacitors. Mxenes and Their Composites.

[41] Pandey R.P., Kallem P., Hegab H.M., Rasheed P.A., Banat F., Hasan S.W. (2022). Cross-linked laminar graphene oxide membranes for wastewater treatment and desalination: A review. J. Environ. Manag..

[42] Liu X., Graham N., Yu W., Shi Y., Sun K., Liu T. (2022). Preparation and evaluation of a high performance Ti_3_C_2_T_x_-MXene membrane for drinking water treatment. J. Membr. Sci..

[43] Wang S., Wang F., Jin Y., Meng X., Meng B., Yang N., Sunarso J., Liu S. (2021). Removal of heavy metal cations and co-existing anions in simulated wastewater by two separated hydroxylated MXene membranes under an external voltage. J. Membr. Sci..

[44] Lin Q., Zeng G., Pu S., Yan G., Luo J., Wan Y., Zhao Z. (2022). A dual regulation strategy for MXene-based composite membrane to achieve photocatalytic self-cleaning properties and multi-functional applications. Chem. Eng. J..

[45] Huang J., Zhang K., Wang K., Xie Z., Ladewig B., Wang H. (2012). Fabrication of polyethersulfone-mesoporous silica nanocomposite ultrafiltration membranes with antifouling properties. J. Membr. Sci..

[46] Al-Araji D.D., Al-Ani F.H., Alsalhy Q.F. (2022). Modification of polyethersulfone membranes by Polyethyleneimine (PEI) grafted Silica nanoparticles and their application for textile wastewater treatment. Environ. Technol..

[47] Al-Timimi D.A.H., Alsalhy Q.F., AbdulRazak A.A., Drioli E. (2022). Novel polyether sulfone/polyethylenimine grafted nano-silica nanocomposite membranes: Interaction mechanism and ultrafiltration performance. J. Membr. Sci..

[48] Shen Z., Chen W., Xu H., Yang W., Kong Q., Wang A., Ding M., Shang J. (2019). Fabrication of a novel antifouling polysulfone membrane with in situ embedment of mxene nanosheets. Int. J. Environ. Res. Public Health.

[49] Zinadini S., Zinatizadeh A.A., Rahimi M., Vatanpour V., Zangeneh H. (2014). Preparation of a novel antifouling mixed matrix PES membrane by embedding graphene oxide nanoplates. J. Membr. Sci..

[50] Kiani S., Mousavi S.M., Saljoughi E., Shahtahmassebi N. (2018). Preparation and characterization of modified polyphenylsulfone membranes with hydrophilic property for filtration of aqueous media. Polym. Adv. Technol..

[51] Qiu S., Wu L., Pan X., Zhang L., Chen H., Gao C. (2009). Preparation and properties of functionalized carbon nanotube/PSF blend ultrafiltration membranes. J. Membr. Sci..

[52] Kiani S., Mousavi S.M., Shahtahmassebi N., Saljoughi E. (2015). Hydrophilicity improvement in polyphenylsulfone nanofibrous filtration membranes through addition of polyethylene glycol. Appl. Surf. Sci..

[53] Díez-Pascual A.M., Díez-Vicente A.L. (2014). Effect of TiO_2_ nanoparticles on the performance of polyphenylsulfone biomaterial for orthopaedic implants. J. Mater. Chem..

[54] Dashtbozorg A., Saljoughi E., Mousavi S.M., Kiani S. (2022). High-performance and robust polysulfone nanocomposite membrane containing 2D functionalized MXene nanosheets for the nanofiltration of salt and dye solutions. Desalination.

[55] Al Aani S., Wright C.J., Atieh M.A., Hilal N. (2017). Engineering nanocomposite membranes: Addressing current challenges and future opportunities. Desalination.

[56] Al-Rashdi B., Somerfield C., Hilal N. (2011). Heavy Metals Removal Using Adsorption and Nanofiltration Techniques. Sep. Purif. Rev..

[57] Zhang Y., Zhang S., Chung T.-S. (2015). Nanometric graphene oxide framework membranes with enhanced heavy metal removal via nanofiltration. Environ. Sci. Technol..

[58] Miao R., Feng Y., Wang Y., Wang P., Li P., Li X., Wang L. (2021). Exploring the influence mechanism of ozonation on protein fouling of ultrafiltration membranes as a result of the interfacial interaction of foulants at the membrane surface. Sci. Total Environ..

[59] Mbareck C., Nguyen Q.T., Alaoui O.T., Barillier D. (2009). Elaboration, characterization and application of polysulfone and polyacrylic acid blends as ultrafiltration membranes for removal of some heavy metals from water. J. Hazard. Mater..

[60] Al-Rashdi B., Johnson D., Hilal N. (2013). Removal of heavy metal ions by nanofiltration. Desalination.

[61] Tian J., Chang H., Gao S., Zhang R. (2020). How to fabricate a negatively charged NF membrane for heavy metal removal via the interfacial polymerization between PIP and TMC?. Desalination.

[62] Zeng G., He Y., Zhan Y., Zhang L., Pan Y., Zhang C., Yu Z. (2016). Novel polyvinylidene fluoride nanofiltration membrane blended with functionalized halloysite nanotubes for dye and heavy metal ions removal. J. Hazard. Mater..

[63] Zhu W.P., Sun S.P., Gao J., Fu F.J., Chung T.S. (2014). Dual-layer polybenzimidazole/polyethersulfone (PBI/PES) nanofiltration (NF) hollow fiber membranes for heavy metals removal from wastewater. J. Membr. Sci..

[64] Gao J., Sun S.-P., Zhu W.-P., Chung T.-S., Chung T.-S. (2014). Chelating polymer modified P84 nanofiltration (NF) hollow fiber membranes for high efficient heavy metal removal. Water Res..

[65] Murthy Z., Chaudhari L.B. (2008). Application of nanofiltration for the rejection of nickel ions from aqueous solutions and estimation of membrane transport parameters. J. Hazard. Mater..

[66] Figoli A., Ursino C., Santoro S., Ounifi I., Chekir J., Hafiane A., Ferjani E. (2020). Cellulose Acetate Nanofiltration Membranes for Cadmium Remediation. J. Membr. Sci. Res..

[67] Hadi S., Mohammed A.A., Al-Jubouri S.M., Abd M.F., Majdi H.S., Alsalhy Q.F., Rashid K.T., Ibrahim S.S., Salih I.K., Figoli A. (2020). Experimental and Theoretical Analysis of Lead Pb^2+^ and Cd^2+^ Retention from a Single Salt Using a Hollow Fiber PES Membrane. Membranes.

[68] Ahmed S.H., Al-Jubouri S.M., Zouli N., Mohammed A.A., Majdi H.S., Salih I.K., Al-shaeli M., Al-Rahawi A.M., Alsalhy Q.F., Figoli A. (2021). Performance Evaluation of Polyethersulfone Membranes for Competitive Removal of Cd^2+^, Co^2+^, and Pb^2+^ Ions from Simulated Groundwater. Geofluids.

[69] Vasyukova I.A., Zakharova O.V., Kuznetsov D.V., Gusev A.A. (2022). Synthesis, toxicity assessment, environmental and biomedical applications of MXenes: A review. Nanomaterials.

[70] Wu J., Yu Y., Su G. (2022). Safety Assessment of 2D MXenes: In Vitro and In Vivo. Nanomaterials.

[71] Karahan H.E., Goh K., Zhang C., Yang E., Yıldırım C., Chuah C.Y., Ahunbay M.G., Lee J., Tantekin-Ersolmaz Ş.B., Chen Y. (2020). MXene Materials for Designing Advanced Separation Membranes. Adv. Mater..

